# Mediterranean diet adherence and sleep pattern: a systematic review of observational studies

**DOI:** 10.1186/s40795-024-00853-x

**Published:** 2024-03-04

**Authors:** Melika fallah, Azadeh Aminianfar, Ahmad Esmaillzadeh

**Affiliations:** 1 Department of Community Nutrition, School of Nutritional Sciences and Dietetics, Tehran University of Medical Sciences, Tehran, Iran P.O. Box 14155-6117,; 2 Research Center for Biochemistry and Nutrition in Metabolic Diseases, Kashan University of Medical Sciences, Kashan, Iran; 3 Obesity and Eating Habits Research Center, Endocrinology and Metabolism Molecular - Cellular Sciences Institute, Tehran University of Medical Sciences, Tehran, Iran; 4 Department of Community Nutrition, Isfahan University of Medical Sciences, Isfahan, Iran

**Keywords:** Mediterranean diet, Sleep pattern, Sleep disorders, Sleep quality, Sleep hygiene, Sleep disturbance, Sleep, Dietary pattern

## Abstract

**Background and aims:**

Despite a huge body of evidence on the linkage between dietary intakes and pattern of sleeping, the findings are controversial. The current study aimed to summarize earlier findings on the association between adherence to Mediterranean diet (MD) and pattern of sleeping.

**Methods:**

This study performed based on PRISMA guideline. Systematically search was applied in PubMed, Scopus and Google Scholar to find out relevant publications appeared up to February 2023. No restrictions on language and time of publication were applied. Duplicate citations were removed. We included observational studies which assessed MD as the main exposure and kind of sleep disorders as the main outcome.

**Results:**

A total of 20 observational studies included. Out of these studies, two were cohort studies and 18 had a cross-sectional design. A total of 21,714 participants included. Usual dietary intakes were assessed using a validated Food Frequency Questionnaire, and a diet history questionnaire. Some studies did not report methods of measuring habitual dietary intakes. Adherence to MD was evaluated by KIDMED questionnaire, PREMED, alternate Mediterranean (aMed) questionnaire, MEDAS questionnaire, MedDietScore, MEDI-LITE score, modified Mediterranean Diet Score (mMDS), Mediterranean food pattern (MFP) and modified Mediterranean diet score (mMED). Pattern of sleeping was examined as sleep quality, sleep duration, sleep latency, sleep efficacy, sleepiness, sleep disturbance, taking a nap and some other sleep disorders.

**Conclusion:**

In conclusion, findings of published studies highlighted the importance of consumption of MD for better sleep quality.

## Intruduction

Sleep is an important physiological function to repair and clear tissue and brain [1]. World Health Organization expressed that 27% of the world’s population suffer from sleep disorders [2, 3]. Other sleep disorders include sleep-disordered breathing (SDB), parasomnias, narcolepsy, and restless leg syndrome [4]. As a common health problem in modern society, sleep disorder have a high incidence in the elderly population, which seriously affect the quality of life and physical and mental health [5]. There is a high prevalence of sleep disturbance worldwide as 7.8% of adults had severe sleep problems in the general population [6]. The prevalence of sleep disorder in older Iranian adults was reported as 48.9% [7]. Medical conditions, depression, anxiety, or cognitive dysfunction can occur along with sleep disorders [8]. These disorders can increase the risk of stroke [9, 10], migraine [11], neurodevelopmental disorders [12] and irritable bowel syndrome [13]. Despite the role of several factors in sleep quality, including age, sex, body weight and depression [14], dietary factors have received great attention recently. Consumption of fruit, vegetables, dairy products and various vitamins and minerals have been previously studied in this regard [15]. However, limited data are available abut theses specific dietary patterns. The Mediterranean diet (MD) is a diet based on high consumption of green leafy vegetables, fruits, fish, healthy fats mainly olive oil, legumes, whole grains, nuts and seeds, moderate intake of dairy products and wine consumption as well as low consumption of processed foods, confectionery and red meat [16, 17]. This diet is low in saturated fat and high in vegetable oils, which observed in Greece and Southern Italy during the 1960s [2]. The MD consists of antioxidants, anti-inflammatory micronutrients and n-3 fatty acids and is characterized by a high intake of monounsaturated fat and fiber [18].

The association between MD and various health-related outcomes has been previously studied [19]. For instance, some documents have shown that the high adherence to the MD can be associated with a lower incidence of chronic diseases and lower aging impairment [20] and frailty [21]. MD also can affect against platelet aggregation [22] and mental disorders including cognitive decline and cancer [23]. In fact, weight loss programs based on the MD, can decrease the lean tissue losses [24]. MD beneficial effects seem to be exerted in both populations of Mediterranean and non-Mediterranean areas [23].

Adherence to the Mediterranean diet has also been studied in relation to sleep patterns; however, findings were conflicting. For example, in a cohort study on the US women aged 20–76 y, adherence to the Mediterranean diet was associated with better sleep quality, higher sleep efficiency, and lower sleep disorders [25]. In contrast, van Egmond et al. in a study on 970 Swedish older men failed to see any significant association between adherence to the MD and self-reported sleep initiation and sleep maintenance problems [26]. Despite having several studies in this field, no systematic review has been conducted on the relationship between MD and sleep disorders. Overall, given the presence of conflicting results on the association between MD and sleep disorders, there is a need for a systematic review summarizing all available findings in this field. Considering above, we performed the current study to systematically review all available studies regard to the relationship between MD and sleep pattern.

## Methods

### Search strategy

This study performed based on PRISMA, protocol for reporting systematic reviews. We performed a comprehensive literature search in the online databases of PubMed, Scopus and Google Scholar up to January 2024. The key words which used for this search were as follows: “Mediterranean Diets” OR “diet” OR “Mediterranean dietary pattern” OR “Feeding Behavior” OR “dietary adherence” OR “dietary score” OR “Mediterranean score OR “MD score” OR “food pattern” OR “dietary habit” OR “Mediterranean dietary score” OR “Mediterranean” OR “dietary pattern” accompanied by “Sleep Disorders” or “insomnia” or “Sleep Wake Disorders” or “Sleep Disorders, Circadian Rhythm” or “Sleep” or “Sleep Deprivation” or “Sleep quality” or “Sleep disturbance” or “Sleep quality index” or “Sleep duration” or “Sleep impairment”. All keywords were based on MeSH and non-MeSH terms. All references of selected articles were also reviewed to find relevant missing publications.

### Selection

**Fig. 1:**
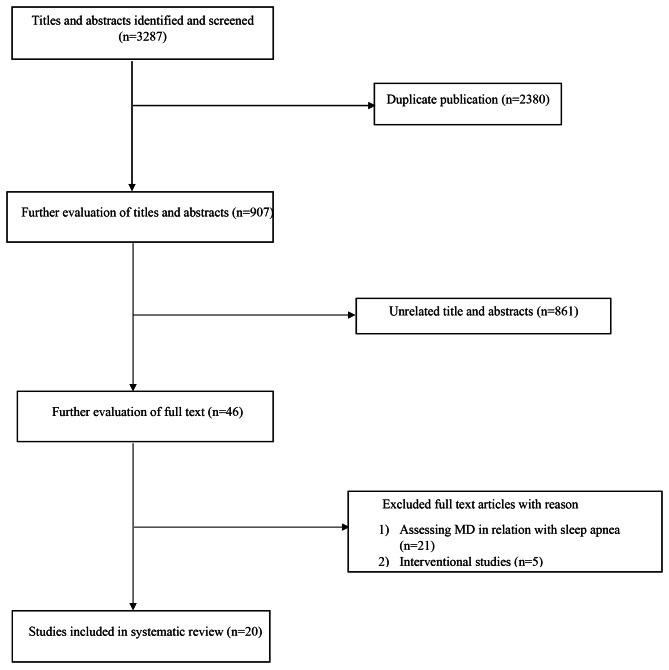
Flow chart of participation

 No restrictions on language and time of publications were applied. Duplicate citations were removed. Observational studies assessing the association between adherence to MD and sleep pattern were included in this systematic review. We included studies with the following criteria: (1) observational studies with prospective, case-control or cross-sectional design; (2) studies that considered adherence to MD as the main exposure; (3) those that had examined every kind of sleep disorders as the main outcome or as one of the outcomes. We excluded letters, comments, short communications, reviews, meta-analyses, ecological studies, and animal studies. A flow diagram of the study selection is shown in Fig. 1.

### Data abstraction

Required data from each eligible study were extracted by two independent investigators, and any disagreements were reconciled by discussion. The following information was extracted: name of the first author, publication year, individuals’ characteristics (mean age and sex), study design, sample size, method of assessment of exposure, type of study outcome, and method of outcome assessment.

## Results

Totally, 3287 articles were found in our initial search; of them 2380 duplicate articles were excluded. After screening the remaining 907 records, 861 unrelated articles were also removed based on title and abstract assessment. Then, 46 articles remained for further evaluation of the full text. Out of those 46 studies, 21 studies were excluded due to assessing MD in relation to sleep apnea and other five studies were also excluded due to having interventional design. Finally, 20 articles were included in the current systematic review. All these studies had examined the association between MD and sleep disorders. Required information for each study has been given in Table 1. All included studies assessed in terms of demographic information as follow including study design, study location and date, exposure assessment, assessment of adherence to Mediterranean diet and outcome assessment.

**Table 1:** Characteristics of included studies on the association between consumption of Mediterranean diet and pattern of sleeping

Author	Gender	Study Design	Country	Age	Outcome Assessment	Exposure Assessment	Sample Size	Study Quality	Outcome	Result	
Zuraitkat et al. 2020	female 100%	Cohort	USA	20–76 y	PSQI ^a^	FFQ^b^ /aMed score	432	Fair quality	Sleep onset latency	lower sleep onset latency	
									Sleep efficiency	no association	
									Sleep disturbances	lower sleep disturbances	
Godos et al. 2019	female 58.5%, male 41.5%	cross sectional	Italy	over 18 y	PSQI	FFQ/Medi-lite score	1936	Good quality	Sleep quality	higher sleep quality	
									Sleep latency	Lower sleep latency	
									Sleep duration	Lower shorter sleep duration	
									Habitual sleep efficacy	lower low sleep efficacy	
									Sleep disturbances	no association	
									Day time dysfunction	lower day dysfunction	
									Self-rated sleep quality	Higher sleep quality	
									need medication to sleep	no association	
van Egmond et al. 2019	females 53%, male 47%	cross sectional	Sweden	71 ± 1	questionnaires	food record/mMDS	970	Good quality	Sleep initiating problems	no association	
									Sleep maintenance problems	no association	
Mamalaki et al. 2018	female 59.2%, male 40.8%	cross sectional	Greece	≥ 65 y	questionnaires	FFQ / MedDietScore	1639	Good quality	Sleep duration	no association	
									Sleep quality	lower trouble falling sleep, higher sleep adequacy	
Flor-Alemany et al. 2020	female 100%	cross sectional	Spain	32.9 ± 4.6	PSQI	FFQ /MFP^c^	150	Fair quality	Sleep quality	higher sleep quality during both the 16th and 34th	
Campanini et al. 2017	female 51.4%, male 48.5%	Cohort	Spain	≥ 60 y	questionnaires/ESS^d^	questionnaires/MEDAS^e^	1596	Fair quality	indicator of Sleep quality	higher sleep quality	
									Sleep duration	lower change in sleep duration	
Castro-Diehl et al. 2018	female 53.6%, male 46.4%	cross sectional	USA	45–84	WHIIRS^f^/Actigraphy	FFQ /aMed score	2068	Good quality	Insomnia	Lower insomnia by no change vs. decrease in a Med score	
									Sleep duration	more likely to sleep 6–7 h/night (vs. <6 h/night)	
Ferranti et al. 2016	female 54.6%, male 45.5%	cross sectional	Italy	11-14y	questionnaires/PDSS^g^	FFQ /KIDMED	1586	Good quality	sleep quality	higher total sleep time, week day sleep time	
									sleep quantity	Lower insomnia	
Adelantado-Renau et al. 2018	girl 48%, boys 52%	cross sectional	Spain	14-18y	PSQI/Wrist-worn accelerometer	KIDMED^h^	269	Fair quality	Sleep quality	Higher sleep quality	
									Sleep duration	no association	
Muscogiuri et al. 2020	females71.5%/male 28.5%	Cross sectional	Italy	51.8 ± 15.7 y	PSQI	PREDIMED	172	Fair quality	Sleep quality	Higher sleep quality	
Boraita et al. 2020	females 50%, males 50%	Cross sectional	Spain	12–17 y	questionnaire	KIDMED	761	Good quality	Sleep duration	More sleep duration	
Rosi et al. 2020	females 46%, males 54%	Cross sectional	Italy	11–14 y	questionnaires/PDSS	KIDMED	409	Good quality	Sleep duration	adequate sleep duration	
									Sleep quality	higher sleep quality	
									daytime sleepiness	Lower sleepiness	
ÖZCAN1 et al. 2021	females 31%, males 69%	Cross sectional	Turkey	19–65 y	PSQI	MEDAS/quesstionnaries	1053	Good quality	Sleep quality	higher sleep quality	
Zaidalkilani et al. 2021	female	Cross sectional	Jordan	36 ± 10 y	AIS	Questionnarie/ PREDIMED	917	Good quality	Insomnia	Lower insomnia	
Gupta et al. 2021	female 26%, male 74%	Cross sectional	Costa Rican		questionnaires	FFQ /aMed score	2169	Good quality	Sleep duration	Adequate sleep duration	
									inconsistent between weekday-weekend sleep	no association	
									take nap	no association	
Bakırhan et al. 2022	females 63%, males 37%	Cross sectional	Turkey	19–64 y	PSQI	MEDAS	250	Good quality	Sleep quality	higher sleep quality	
Naja et al. 2022	Females 81.5%, males 18.5%	Cross sectional	Emirates	22 y	PSQI	KIDMED	503	Good quality	Sleep quality	better subjective sleep quality	
									sleep latency	less sleep latency	
									sleep disturbance	less sleep disturbance	
									Sleep medication	No association	
									Sleep duration	No association	
									daytime dysfunction	less daytime dysfunction	
									Sleep efficacy	No association	
Yaghtin et val. 2022	female	Cross sectional	Iran	12–18 y	ISI ^j^	FFQ/mMED ^k^	733	Good quality	Insomnia	Lower insomnia	
López-Gil et al. 2023	Females 55.3%, males 44.7%	Cross sectional	Spain	12–17 y	questionnaire	KIDMED	847	Good quality	Sleep duration	Higher sleep duration	
Mantzorou et al. 2023	Females 48.4%, males 51.6%	Cross sectional	Greek	≥ 65 y	PSQI	MedDietScore/FFQ	3254	Good quality	Sleep quality	Higher sleep quality	

a. Pittsburgh Sleep Quality Index

b. Food Frequency Questionnaire

c. Mediterranean food pattern

d. Epworth Sleepiness Scale

e. Mediterranean Diet Adherence Screener

f. Women’s Health Initiative Insomnia Rating Scale

g. Parkinson’s disease sleep scale

h. Mediterranean Quality Index for children and teenagers

i. Athens Insomnia Scale

j. Iranian version of the Insomnia Severity Index

k. modified Mediterranean diet score

## Demographic information of included studies

### Study design

Totally, out of 20 included studies, two were cohort studies [25, 27] and eighteen had cross-sectional design [26, 28–36]. The duration of follow up for two cohort studies ranged from one year to 2.8 years [25, 27].

### Participant characteristics

Included studies consisted 21,714 participants. Sample sizes across studies varied from 150 [31] to 3254 people [37]. Participants aged over 11 years old. All studies were on both gender, except for four articles that were performed on women [25, 31, 38, 39]. Five studies were on older adults (60–84 y) [26, 27, 29, 33, 37] and seven on young adolescents (11–15 y) [28–30, 35, 38, 40, 41]. All studies had assessed healthy people, except for of study of Castro-Diehl et al. [29] that had included atherosclerosis patients. One study had included pregnant women [31].

### Study location and date

Out of 20 studies, two were carried out in the USA [25, 29], four in Italy [30, 32, 34, 35], five in Spain [27, 28, 31, 40, 41], two in Turkey [42, 43], two in Greece [33, 37] and one each in Sweden [26], Iran [38], Jordan [39], Emirate [44] and Costa Rica [36]. All studies were published between 2016 and 2023.

## Exposure and outcome assessment

### Exposure assessment

Evaluation of usual dietary intakes were done in the included studies as follow: nine studies used validated Food Frequency Questionnaire (FFQ) [25, 29–33, 36–38] to assay dietary intakes. Dietary records [26] and diet history [27] was in tow other studies. The remaining three studies did not report any data on the methods of measuring habitual dietary intakes [28, 34, 35, 39, 41–44].

### Assessment of adherence to MD

Calculation of Mediterranean diet score was done by the following methods in the included studies: a validated 16 items KIDMED questionnaire was used in six studies [28, 30, 35, 40, 41, 44] and a 14 items validated PREMED Questionnaire was applied in two studies [34, 39]. Three studies mentioned a 10 items validated alternate Mediterranean (aMed) questionnaire [25, 29, 36]. In addition, three studies used 12 items validated MEDAS questionnaire [27, 42, 43]. The others used 11 items Med Diet Score [33, 37], 9 items MEDI-LITE score [32] and 8 items modified Mediterranean Diet Score (mMDS) [26, 38]. The study of Flor Alemany et al. had used Mediterranean food pattern (MFP) which was a validated questionnaire [31].

### Outcome assessment

Sleep quality and quantity were separately considered as primary outcomes. In general, eleven studies assessed sleep quality [27, 28, 31–35, 37, 42–44], ten studies considered sleep duration [27–29, 32, 33, 35, 36, 40, 41, 44], three studies investigated sleepiness [30, 33, 35], four studies investigated sleep disturbances [25, 31, 32, 44], three studies examined taking nap [30, 33, 36] and three studies examined sleep efficacy and sleep latency [25, 32, 44]. All other sleep disorders that were assessed in two other studies were need medication to sleep [32, 44], day time dysfunction [32, 44], sleep initiating problems [26], sleep maintenance problems [26], not quiet sleep, awaken short of breath or with a headache, feel drowsy or sleepy during the day, trouble falling asleep, awaken during sleep and have trouble falling asleep, trouble staying awake during the day, snore during sleep, snoring and sleep adequacy [33], insomnia [29, 38, 39], inconsistent between weekday-weekend sleep [36] bed time and wake time on weekday and weekend, total sleep time, weekdays sleep time and weekend sleep time [30]. To examine sleep disorders, Pittsburgh Sleep Quality Index (PSQI), a 19 items validated questionnaire, was used by nine studies [25, 28, 31, 32, 34, 37, 43, 44], Women’s Health Insomnia Rating Scale (WHIIRS) and a five-items validated questionnaire, was used in the study of Castro-Diehl et al. [29]. Iranian version of the Insomnia Severity Index (ISI) and Athens Insomnia Scale (AIS( was used to assess insomnia in two studies [38, 39]. A validated self-report questionnaires and pediatric daytime sleepiness scale (PDSS) was used by two studies [30, 35]. An Epworth Sleepiness Scale (ESS), an eight items validated questionnaire, was used in the study of Campanini et al. [27] Some other questionnaires were used in the other eight remaining studies [26, 27, 30, 33, 35, 36, 40, 41]. Actigraphy [29] and Wrist-worn accelerometer [28] were used for sleep duration assessment in just two studies, which allowed for evaluation of sleep phenotype.

## The association between MD and sleep disorders

Sleep disorders in relation to the adherence to the Mediterranean diet were investigated in different studies as follow:

### Sleep quality

Sleep quality was reported in the most included studies. Mamalaki et al. [33] assessed sleep quality by examining following items: daytime sleepiness, sleep adequacy, sleep disturbance, trouble falling asleep, take naps, feel drowsy or sleepy during the day, trouble staying awake during the day, snoring, snore during sleep, awaken during sleep and have trouble falling asleep and awaken short of breath or with a headache. Ferranti et al. [30] reported the association of a component of sleep quality with MD and did not consider total sleep quality. Eleven studies that evaluated the association of MD with sleep quality reported a greater adherence to the MD in association with a better sleep quality [37, 42–44].

### Sleep duration

Seven publications assessed MD in relation to sleep duration. Six studies reported a significant association between adherence to MD and sleep duration [27, 32, 35, 36, 40, 41]. While three studies reported no significant association between MD and duration of sleep [28, 33, 44]. Castro-Diehl et al. [29] compared moderate-high aMed score to a low aMed score across different categories of objectively measured sleep duration (< 6 h/night, 6–7 h/night, 7–8 h/night and > 8 h/night). They reached no significant association in total; however, participants with a moderate-high aMed score were more likely to sleep 6–7 h/night than those who had a low aMed score.

### Sleep latency

Sleep latency was examined in three studies [25, 32, 44]. All of them had reported adherence to the MD was associated with a lower sleep latency.

### Sleep efficacy

Based on three studies that assessed adherence to MD in relation to sleep efficacy, two of them reported no significant association [25, 44] and the other found a lower occurrence of low sleep efficacy by a greater adherence to MD [32].

### Sleepiness

All three studies [30, 33, 35] that evaluated adherence to MD and sleepiness by PDSS [35] and self-report questionnaires [30, 33] reported an inverse association between sleepiness and MD.

### Sleep disturbances

Four studies considered sleep disturbances as the outcome of interest. Zuraitkat et al. [25] and Naja et al. [44] found an inverse association between consumption of MD and sleep disturbances among women, however, no significant association was seen between sleep disturbances and MD in Godos et al. [32] and Mamalaki et al. [33] studies.

### Taking a nap

All three studies that evaluated the association between adherence to MD and taking a nap reported no statistically significant associations [30, 33, 36].

### Other sleep disorders

In addition to the disorders mentioned above, some other sleep disturbances were also examined in some studies. Needing medications to sleep and self-rated sleep quality [32] as well as sleep initiating problems and sleep maintenance problems [26] were also assessed in any other investigation. In general, only self-rated sleep quality was positively associated with MD. Other sleep outcomes were not associated with this dietary pattern. Mamalaki et al. [33] found no significant association between adherence to MD and some sleep disorders including: not quiet sleep, awaken short of breath or with a headache, feel drowsy or sleepy during the day, trouble falling asleep, awaken during sleep and have trouble falling asleep, trouble staying awake during the day, snore during sleep, snoring and sleep adequacy. However, they found lower occurrence of trouble falling sleep and higher sleep adequacy among those with the greatest adherence to MD than those with the lowest adherence. In the study of Castro-Diehl et al. [29], the association between three-levels (no change, decreased and increase) of change in aMed score was examined in relation to insomnia. No change in aMed score in almost a ten year period, compared with a decreased aMed score in this period, was associated with improved insomnia. Although a significant association was seen between adherence to MD with total sleep time and weekdays sleep time, no significant association was reported between sleep time on weekend and bed time or wake time on weekday and weekend with MD in study of Ferranti et al. [30].

### Study quality

The quality of studies included in the current review was assessed using the Newcastle Ottawa Scale (NOS), designed for nonrandomized studies [45]. According to this scale, a maximum of 9 points awarded to each cohort study according to the following parameters: 4 points for selection of participants, 2 points for comparability, and 3 points for the assessment of outcomes. A maximum of 10 points awarded to each cross sectional study include: 5 points for selection of participants, 2 points for comparability, and 3 points for the assessment of outcomes. A study with score from 7 to 9 has high quality, 4–6, has high risk, and 0–3 has very high risk of bias. Based on NOS scoring, we found that 15 studies had high quality [26, 29, 30, 32, 33, 35, 36], four studies had a high risk of bias [25, 27, 28, 34] and one study [31] had a very high risk of bias.

## Discussion

In this study, we summarized earlier studies about the adherence to MD and sleep quality. Summarizing previous findings, we found that adherence to MD might help sleeping better. Sleep disturbance can be linked to poor health outcomes and increase the risk of developing metabolic disease and cardiovascular events [46]. Nutrition can profoundly affect the hormones and inflammation which directly or indirectly contribute to good or bad sleep quality [47]. MD has long been studied in relation to several health related outcomes including sleep hygiene [47]. The MD was first defined as a diet with low saturated fats and high vegetable oils [48] with a particular focus on extra virgin olive oil. It contains high amounts of vegetables including leafy green vegetables, fruits, cereals, nuts and pulses/legumes, moderate intakes of fish and other meat, dairy products and red wine, and low intakes of eggs [47, 49]. It has been supposed that MD can increase the secretion of brain derived neurotrophic factors and improve total body antioxidant capacity [50]. Polyphenols intake in this dietary pattern can help explaining its Anti-inflammatory and antioxidant properties, through which it might affect learning and memorizing [51–53]. MD contains dietary sources of tryptophan, which is an amino acid that is associated with improving sleep quality [52]. Consumption of MD is accompanied with less sleep disorders because of its specific dietary components as high levels of very long-chain n-3 PUFAs [54] that might induce secretion of melatonin and serotonin with their fundamental role in better sleep quality which modulates circadian rhythm [50, 55]. MD can also improve adiposity and body weight, blood pressure, blood lipids, glucose metabolism and insulin sensitivity that may beneficially affect brain function, cognition, and mood which are also important to sleep [56, 57]. Moreover, it is considered that gut microbiota mediate sleep effects of the MD as some of them can promote higher production of SCFA and serotonin, and improve oxidative stress, inflammation, neurologic, and cognitive functions [58, 59]. In addition, olive oil favorably changed gut microbiota composition and metabolic function, maybe by increasing SCFA production [58].

We were not able to do a meta-analysis on these studies because of heterogeneity between studies in terms of reporting different effect sizes. In addition, the study design in included studies were different, which prohibited us again to derive a quantitative assessment of available literature. Most included studies, except for two, did not use actigraphy and Wrist-worn accelerometer to measure sleep quality, which are non-subjective method for assessment of sleep quality. Others had mostly used self-reported questionnaires to examine sleep quality, which are subject to recall and social biases. About assessing dietary pattern all studies had used self-reported questionnaires. Therefore, misclassification of study participants is unavoidable. The generalizability of our findings should be done cautiously because most included studies had examined a particular age group or had limited the analysis to one gender. Our quality assessment of included studies indicated that out of 12 studies, only seven studies had a high quality and the five remaining studies had a high risk or a very high risk of bias, which might further limits the reliability of findings in earlier studies in this regard.

In conclusion, most findings of published studies highlight the importance of consumption of MD for better sleep quality. Given the different forms of sleep pattern examined in the earlier studies, future large-scale, international, multicenter, population-based, epidemiological studies with samples from different areas like as other countries worldwide, urban, rural and island regions, are essential for more reliable conclusions. Clinical intervention studies to examine the effect of consumption of MD on sleep quality are needed. More animal studies can provide a better view of the mechanisms mediating the association between MD and sleep features. In addition, objective neurophysiological tools for sleep assessment (for example actigraphy, polysomnography) are suggested to widely use in feature studies. Moreover, other studies could examine the effects of meal timing and frequency, in the relation of the MD and sleep quality and quantity. Face-to-face interviews with validated questionnaire such as PSQI is recommended in future studies to reduce recall bias and to increase the validity of the responses. By further studies have been done on these desired variables, meta-analysis would be allowed to be performed. Therefore we can obtain more accurate information and make stronger recommendations for dietary pattern and sleep hygiene.

## Data Availability

All data generated during this study are included in this published article.
